# Refractory Bleeding From an Ileal Conduit in a Patient With Antiphospholipid Syndrome: A Multidisciplinary Management Challenge

**DOI:** 10.7759/cureus.88881

**Published:** 2025-07-28

**Authors:** Asmita Hossain, Toby Murray, Katherine Wise, Ra'ed Haddad

**Affiliations:** 1 Urology, Surrey and Sussex Healthcare NHS Trust, Redhill, GBR; 2 Urology, St George’s Hospitals NHS Foundation Trust, London, GBR

**Keywords:** anticoagulation, antiphospholipid antibody syndrome (aps), computed tomography (ct) angiogram, ct urogram, gastroenterology and endoscopy, ileal conduit, multidisciplinary management, percutaneous nephrostomy tube, recurrent hematuria

## Abstract

Bleeding from an ileal conduit is a rare but potentially life-threatening complication, typically associated with portal hypertension and stomal varices. However, intra-conduit hemorrhage in benign settings is exceedingly rare. We report a case of a 54-year-old woman with a complex history including antiphospholipid syndrome (APS), who presented with persistent bleeding from an ileal conduit, ultimately requiring multidisciplinary intervention after failed conservative management. This case illustrates the challenges of balancing anticoagulation in high thrombotic risk patients and the importance of timely escalation to specialist care.

## Introduction

Ileal conduits are a common form of urinary diversion following cystectomy. While complications such as infection and stomal stenosis are well-documented, hemorrhage is uncommon, particularly from within the conduit itself. Most reported cases originate from the stomal margins and are related to portal hypertension or local trauma [1,2]. Bleeding within the conduit in patients without underlying malignancy is exceptionally rare. Xu et al. described stomal variceal hemorrhage in the context of portal hypertension [1], and Pietrzak et al. reported a case of life-threatening hemorrhage from an ileal conduit requiring emergency intervention [2]. However, intra-conduit bleeding without malignancy or varices is sparsely documented. 

Antiphospholipid syndrome (APS) is a systemic autoimmune disorder characterized by the presence of antiphospholipid antibodies (aPL), including lupus anticoagulant, anticardiolipin antibodies, and anti-β2 glycoprotein I. These antibodies contribute to a prothrombotic state by activating endothelial cells, increasing tissue factor expression, interfering with natural anticoagulant pathways, and promoting platelet aggregation [3,4]. Clinically, APS is associated with recurrent arterial and venous thromboses, as well as pregnancy-related complications. However, bleeding manifestations can also occur. These are often linked to moderate to severe thrombocytopenia, small-vessel vasculitis, or the effects of long-term anticoagulation therapy [3]. Additionally, the presence of lupus anticoagulant may prolong activated partial thromboplastin time (APTT), complicating coagulation monitoring and increasing the risk of therapeutic imbalance. Inflammation of the vascular endothelium and microangiopathy have also been suggested as contributors to mucosal or soft-tissue bleeding in APS [4]. We present a case in which APS likely played a central role in the development of refractory hematuria from an ileal conduit, requiring multidisciplinary management despite the absence of malignancy or variceal disease.

## Case presentation

A 54-year-old woman with a long-standing history of neuropathic bladder underwent cystectomy and ileal conduit formation at the age of 16. Her medical background included APS, cardiomyopathy, chronic kidney disease stage 3, atrophic left kidney, hypertension, type 2 diabetes mellitus, and recurrent pulmonary emboli requiring long-term therapeutic enoxaparin. 

**Figure 1:**
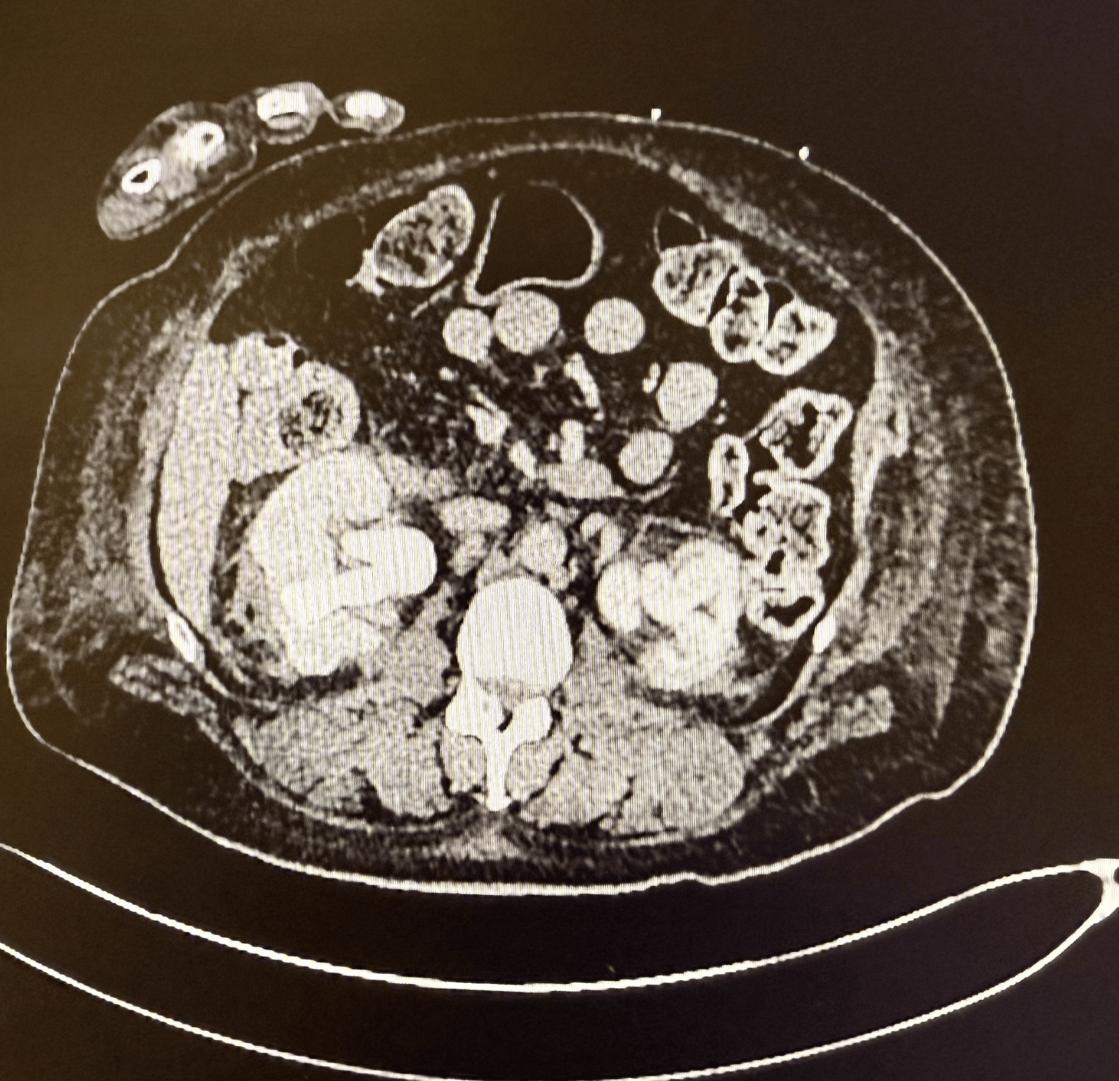
The CT urogram showed right perinephric stranding; no bleeding source was noted.

She presented with a one-week history of visible hematuria from her urostomy bag, which had acutely worsened in the previous 24 hours with large clots and reduced urinary output. She also complained of abdominal pain and nausea, but remained afebrile. Initial blood investigations showed hemoglobin of 132 g/L, white cell count (WCC) of 7x109/L, and an estimated glomerular filtration rate (eGFR) of 37 mL/min (Table 1). A CT urogram did not demonstrate any obstructive uropathy or active bleeding, but right-sided perinephric stranding and left-sided small kidney were noted (Figure 1), raising the possibility of pyelonephritis. Anticoagulation was held, and empirical intravenous co-amoxiclav was started. 

**Table 1:** Key laboratory investigations

Parameter	Result range	Observed results
Hemoglobin	120–160 g/L (adult female)	132 → 119 → 75 → 88 (post transfusion)
White cell count	4.0–11.0 ×10⁹/L	7.0 → 11.3 → 14.3
Sodium	136–145 mmol/L	139 → 139 → 140 → 140
Potassium	3.5–5.1 mmol/L	4.5 → 4.0 → 4.0 → 3.9
Urea	2.1–8.0 mmol/L	7.4 → 9.1 → 9.0 → 7.8
Creatinine	45–90 µmol/L	165 → 189 → 175 → 165
Estimated glomerular filtration rate	≥ 60 mL/min (normal); chronic kidney disease present ≤ 60	37 → 30 → 28 → 30
C-reactive protein	<5 mg/L	54 → 123 → 206
International normalized ratio	0.9–1.2 (normal)/therapeutic as needed	1.3 (at presentation)

Within the following 48 hours, her condition deteriorated with increased hematuria, clot retention, and a decline in hemoglobin to 119 g/L. Her C-reactive protein (CRP) rose to 54 mg/L, WCC to 11.3 ×10⁹/L (Table 1), and she became hypotensive (blood pressure (BP) 87/59 mmHg). A large obstructive clot was removed manually, followed by a significant hematuria gush. Despite initial improvement, her hemoglobin dropped further to 75 g/L, prompting transfusion of one unit of packed red cells. 

**Figure 2:**
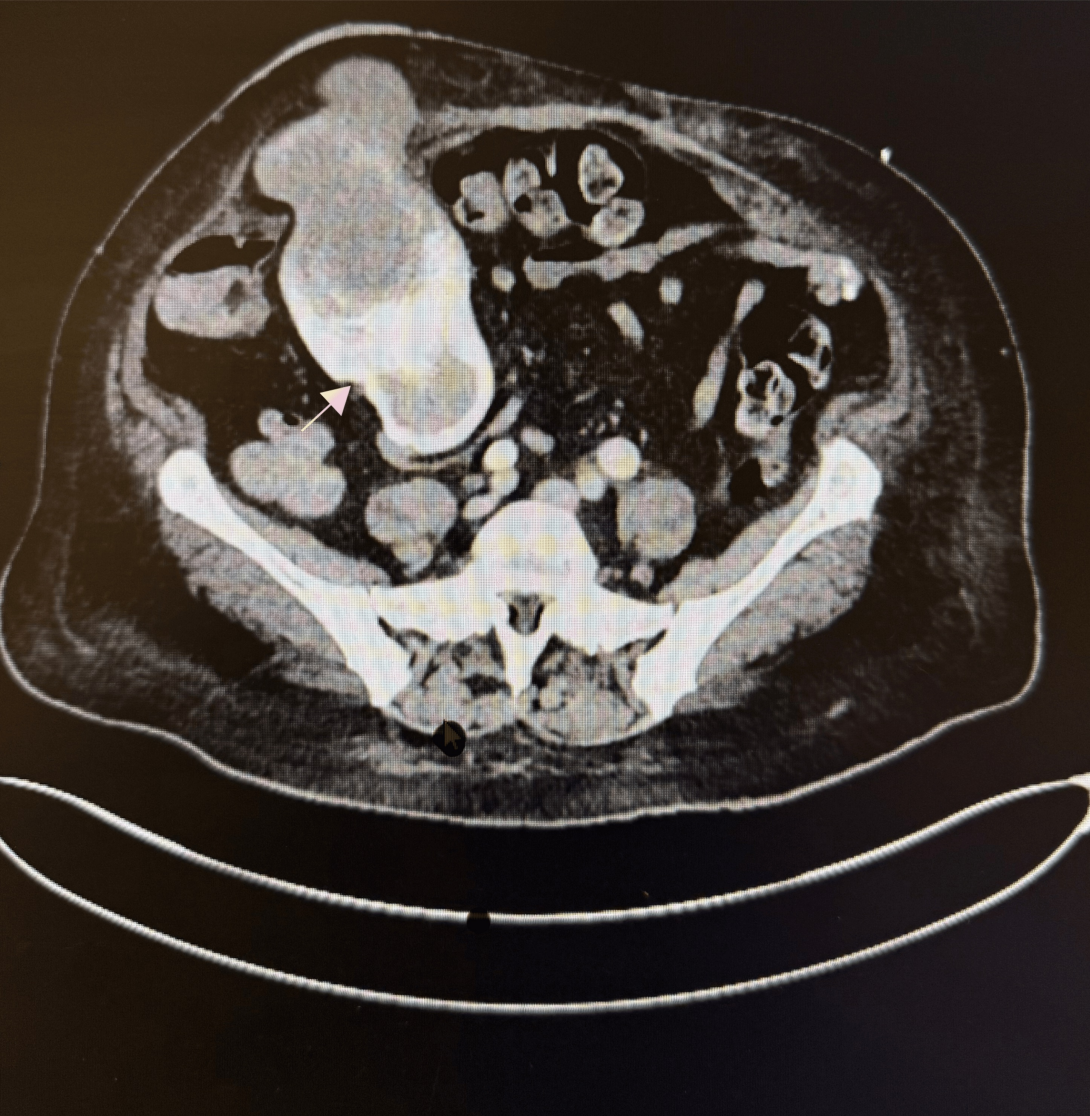
CT angiography confirmed active contrast extravasation within the ileal conduit.

On day 3, CRP rose to 123 mg/L and WCC to 14.3 ×10⁹/L (Table 1); antibiotics escalated to meropenem. CT angiography (Figure 2) confirmed contrast extravasation in the conduit. During an acute confusion episode, a Medical Emergency Team (MET) call was activated, and the brain CT was normal. Hematology recommended switching to unfractionated heparin (5,000 U twice daily (BID)) and permitted tranexamic acid (TXA) in view of her raised activated partial thromboplastin time (APTT) from lupus anticoagulant. 

Multiple attempts to secure local interventional radiology (IR) support were unsuccessful due to high procedural risk. After multidisciplinary consultation with hematology, the IR team, gastroenterology, and urology teams, a decision was made to transfer the patient to a tertiary urology center for specialist input. 

**Figure 3:**
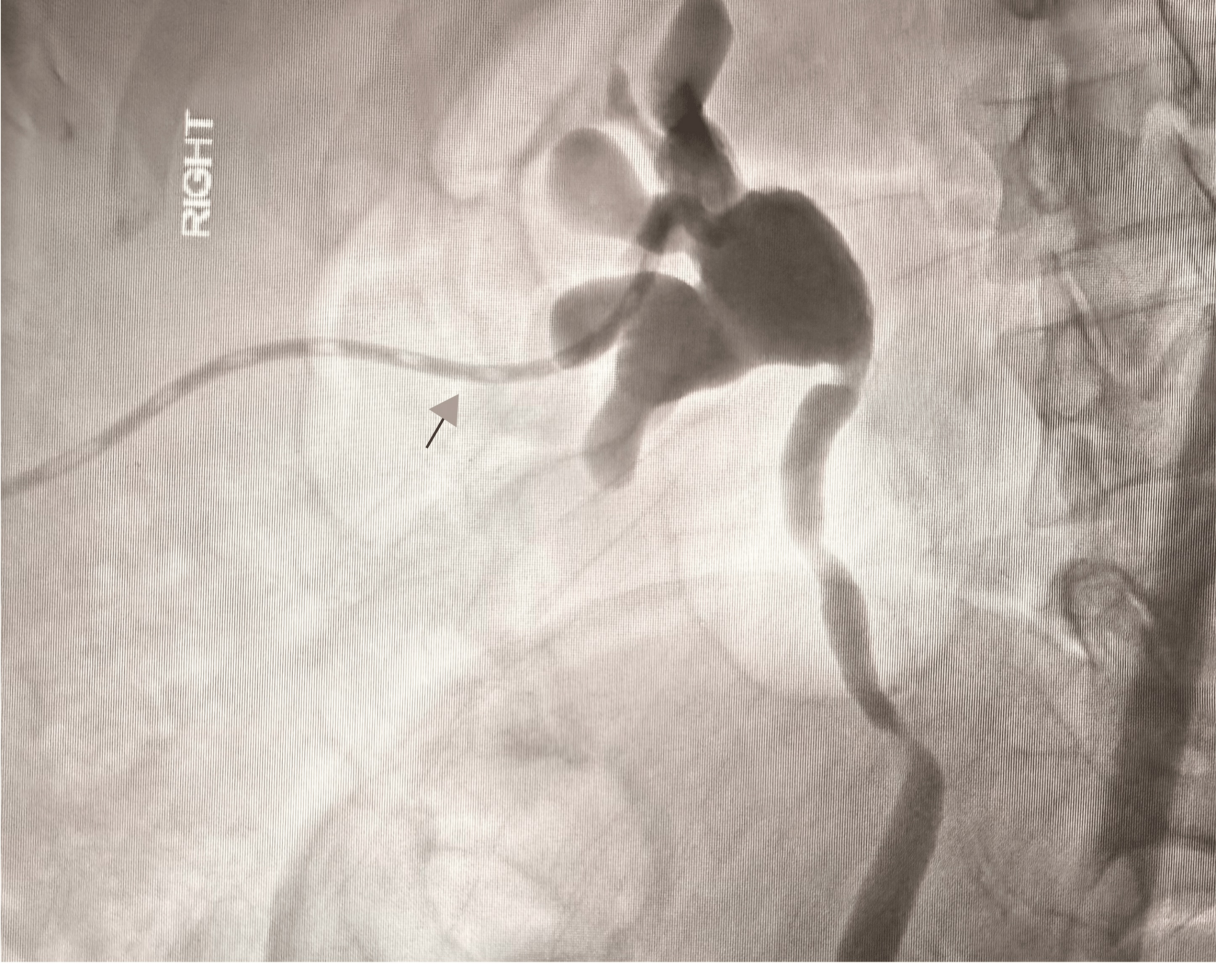
Nephrostogram showed a right percutaneous nephrostomy tube in situ

Upon transfer, repeat CT revealed persistent contrast pooling in the conduit and bilateral hydronephrosis, suggestive of clot retention. A right nephrostomy was inserted to decompress the system and protect the solitary functioning kidney (Figure 3). Two weeks later, a follow-up ultrasound confirmed resolution of hydronephrosis and preserved renal perfusion. 

At the tertiary center, flexible conduitoscopy identified a superficial mucosal bleeder with an adherent clot near the conduit wall. Hemostasis was achieved using Bugbee diathermy, and a 16 French two-way catheter was inserted for tamponade. She remained stable on unfractionated heparin and was discharged with a course of co-amoxiclav and a personalized anticoagulation plan under hematology guidance. Table 2 presents a timeframe of clinical events and management strategies.

**Table 2:** Timeframe of clinical events and management strategies

Timeframe	Clinical event and management
Admission day	Presented with visible hematuria from an ileal conduit. Baseline labs and initial assessment completed.
Day 1	CT urogram performed; no active bleeding source identified; empirical IV antibiotics initiated.
Day 2	Hemoglobin dropped; manual clot removal performed; patient became hypotensive; one unit of red blood cells (RBC) transfused.
Day 3	Inflammatory markers worsened; CT angiography revealed active bleeding; Medical Emergency Team (MET) call due to confusion.
Day 4	Local interventional radiology (IR) support unavailable; decision made to transfer to a tertiary urology centre.
Day 5	Transfer completed; repeat CT showed contrast pooling in the conduit and bilateral hydronephrosis.
Day 6	Right nephrostomy inserted to decompress the system.
Week 2	Flexible conduitoscopy performed; identified superficial mucosal bleeder; hemostasis achieved via Bugbee diathermy and tamponade catheter inserted.
Mid-week 2	Follow-up ultrasound confirmed resolution of hydronephrosis and preserved renal perfusion.
End of week 2	Patient discharged with antibiotic course and personalized anticoagulation plan in place.

## Discussion

Ileal conduit, first introduced by Bricker in the 1950s, remains the most widely used form of incontinent urinary diversion following cystectomy for both benign and malignant conditions, with continent alternatives such as Indiana pouches and orthotopic neobladders also commonly employed in suitable patients [5]. Early complications of ileal conduits include urine leakage, wound dehiscence, stomal infection, and ileus, while late complications occurring beyond 90 days consist of stomal stenosis, conduit strictures, urinary tract infections (UTIs), urolithiasis, and upper-tract deterioration such as hydronephrosis [6, 7].

Although hemorrhage is infrequent, it remains a serious complication. Stomal and conjugated variceal bleeding, often secondary to portal hypertension, are among the most commonly reported sources [1, 2]. These typically necessitate interventional radiology with embolization or surgical shunting [1-3]. Rare causes of intra-conduit hemorrhage include mucosal ulceration, trauma, calculi, infection, and recurrent malignancy [2, 6].

In our patient, however, intra-conduit bleeding occurred in the absence of varices or malignancy. Instead, APS, an autoimmune condition characterized by aPL antibodies, likely played a key role. APS is conventionally associated with thrombotic events, including deep vein thromboses and strokes; yet paradoxical bleeding can occur due to small-vessel vasculopathy, thrombocytopenia, or effects of anticoagulation therapy [3, 8]. Indeed, cases of gastrointestinal bleeding related to APS have been documented [8, 9], though bleeding from an ileal conduit has not been previously reported.

The management of APS typically involves lifelong vitamin K antagonists with a target international normalized ratio (INR) of 2.0-3.0 for venous thrombosis and higher (3.0-4.5) for arterial events; low-dose aspirin may be used adjunctively in high-risk scenarios [10]. However, in the setting of active bleeding, anticoagulation must be carefully balanced: in our case, unfractionated heparin was initiated to allow easy reversal if needed, and TXA was used to promote coagulation.

Despite cessation of anticoagulation, IV antibiotics, and volume resuscitation, bleeding persisted. CT angiography identified active extravasation into the conduit. Transfer to a tertiary center enabled the use of endoscopic Bugbee diathermy, which successfully achieved hemostasis.

This case broadens the etiological spectrum of ileal conduit bleeding, highlighting APS as a potential cause. It also emphasizes that minimally invasive endoscopic intervention within a multidisciplinary framework can be effective, especially when portal hypertension-related strategies are inappropriate.

Learning points

Ileal conduit bleeding is extremely rare unless predisposing factors are present, such as coagulopathy, infection, or vascular abnormalities. Conservative management often fails in significant conduit bleeding; prompt recognition and escalation are essential. Multidisciplinary discussion involving hematology, gastroenterology, urology, radiology, and intensive care should occur early, especially in patients on anticoagulation. Timely decisions for operative or endoscopic control of bleeding can prevent further morbidity, particularly in complex cases such as those with APS. Flexible conduitoscopy and endoscopic diathermy can be effective in controlling superficial bleeding within the conduit. 

## Conclusions

Ileal conduit bleeding, particularly intra-conduit hemorrhage in benign conditions, is rare but serious. In patients with complex hematological disorders such as APS, early multidisciplinary involvement, timely imaging, and escalation to tertiary care are essential. This case illustrates the importance of tailoring anticoagulation, recognizing when conservative measures have failed, and coordinating specialist care to ensure optimal outcomes.
